# A Brief History of Adherons: The Discovery of Brain Exosomes

**DOI:** 10.3390/ijms21207673

**Published:** 2020-10-16

**Authors:** David Schubert

**Affiliations:** Salk Institute for Biological Studies, La Jolla, CA 92037, USA

**Keywords:** exosomes, extracellular matrix, heparan sulfate proteoglycans, neurotrophic factors

## Abstract

Although exosomes were first described in reticulocytes in 1983, many people do not realize that similar vesicles had been studied in the context of muscle and nerve, beginning in 1980. At the time of their discovery, these vesicles were named adherons, and they were found to play an important role in both cell–substrate and cell–cell adhesion. My laboratory described several molecules that are present in adherons, including heparan sulfate proteoglycans (HSPGs) and purpurin. HSPGs have since been shown to play a variety of key roles in brain physiology. Purpurin has a number of important functions in the retina, including a role in nerve cell differentiation and regeneration. In this review, I discuss the discovery of adherons and how that led to continuing studies on their role in the brain with a particular focus on HSPGs.

## 1. Introduction

All cells have, to varying extents, physical contact with the extracellular space surrounding them. This may be proximal cells, the fluid components of blood, or, in most cases, extracellular matrix (ECM). In the brain, the ECM constitutes a significant part of its mass, but compared to other aspects of brain physiology, relatively little is known about its structure, assembly, and function. This was certainly true in 1970 when I had just finished my Ph.D. and started, with Steve Heinemann, the neuroscience program at the Salk Institute in La Jolla. My thesis with Melvin Cohn, a Founding Fellow at Salk, was on the ultimate protein secretory cells—antibody-producing B cells. The work in the Cohn lab and many other immunology labs was largely based upon tissue culture studies of immune cells, and it was very successful. Because the use of cultured cells was an established paradigm in immunology, the approach of my lab in the new neuroscience department was to use cultured cells of both neuronal and non-neuronal origin to identify proteins involved in cellular differentiation and function.

For example, we both made and used myoblast cell lines from skeletal muscle to study synapse formation as well as the popular H9 cardiomyocyte cell line. Over the years, these cells have been distributed around the world and used for a variety of purposes, including the isolation of the first oncogene [1]. These cells secrete many growth factors, such as glial-derived neurotrophic factor (GDNF) that was used to treat Parkinson’s disease [2], and cell differentiation factors [3]. Importantly, studies on these cells led to early insights into exosomes, extracellular matrix formation, and cell adhesion in the nervous system, which is the subject of this brief review.

One purpose in making the clonal cell lines from the brain was to be able to study the nature and function of molecules involved in neural development and physiology, such as synapse formation. We had previously established the first neuronal cell line and shown that it could make synapses with itself and with skeletal muscle, so the proof of concept was there [4]. However, additional cell lines from the brain were required. The approach we used was to inject nitroethylurea (NEU) into pregnant rats and isolate and clone cell lines from the resultant brain tumors. This program was successful and yielded about 100 distinct cell lines, of which five were neuronal as defined by an electrically excitable membrane and neurotransmitter synthesis [5,6]. The next step was to determine the repertoire of proteins that the different types of cells made and then use this information to study brain tissue in vivo, perhaps via the use of antibodies produced against the proteins. As a start, we examined the proteins secreted by some of the nerve and glia lines using the primitive tools of the day—mainly sodium dodecyl sulfate (SDS) polyacrylamide gels [7]. Although at the time, we could not identify individual proteins, it was observed that a large amount of the secreted proteins adhered to cell culture dishes. This material that we termed substrate-attached material (SAM) was clearly a subset of the total secreted material and appeared to be involved in promoting the adhesion of the cells to culture dishes [7]. Curiously, growth factors, such as nerve growth factor (NGF) and insulin, increased cell–substratum adhesion, in part, by modifying the composition of SAM [8]. This led to the question of what was the composition of SAM, which we considered an in vitro surrogate of ECM, and how was it delivered to the extracellular space?

## 2. Cell Adhesion Is Dependent Upon High Molecular Weight Protein Complexes

**Figure 1:**
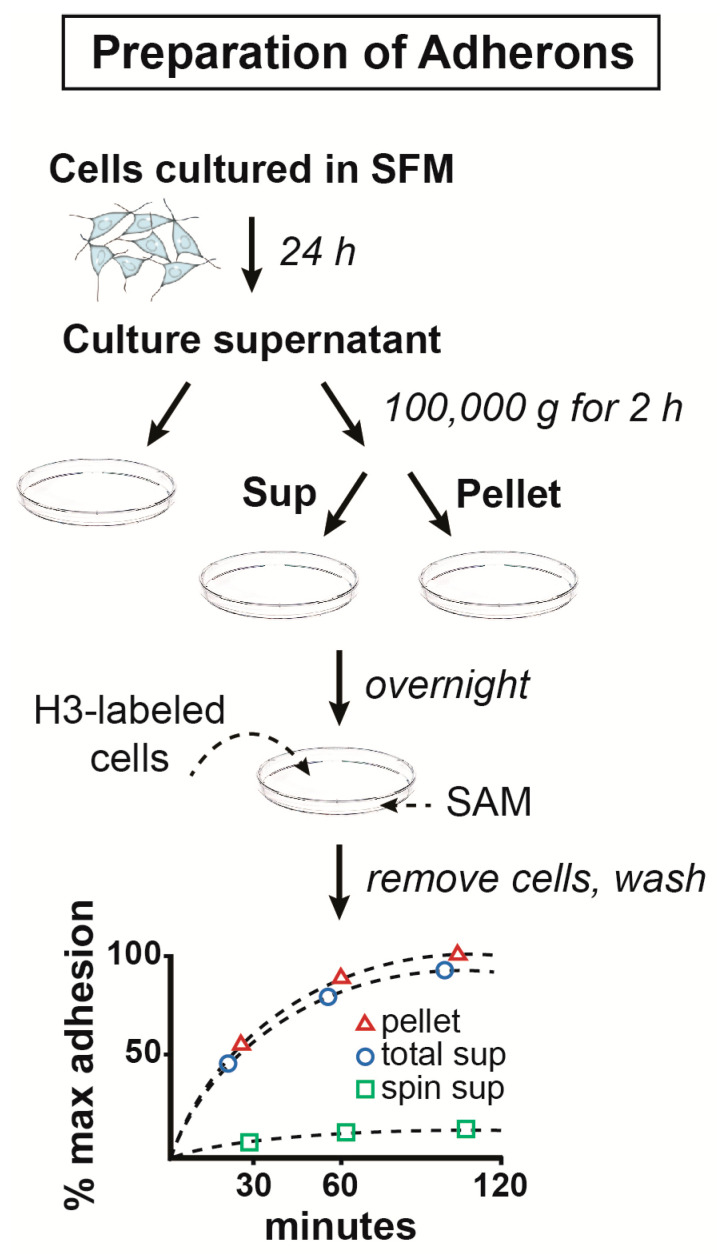
Approach for the preparation of adherons and analysis of their role in cell–substratum adhesion.

To study the role of secreted molecules in cell–substratum adhesion, we devised a very simple cell-SAM binding assay [9]. Cells do not stick to non-tissue culture-coated plastic petri dishes but do stick when the surface is exposed overnight to conditioned medium from cells grown in serum-free medium for 24 h (Figure 1). Cells were labeled with ^3^H-leucine and then manually dissociated (or, in some cases, non-adherent cells were used) and then were added to uncoated or coated petri dishes in serum-free 4-(2-hydroxyethyl)-1-piperazineethanesulfonic acid (HEPES) medium with glucose, and the rate of adherence determined by harvesting individual dishes as a function of time and quantifying the radioactivity. In most cases, the binding curve flattened after about 1 h in the presence of excess cells (Figure 1), suggesting that SAM binding sites were saturated. Additionally, the serum-free conditioned medium could promote cell–cell adhesion. However, when the serum-free conditioned medium was centrifuged at 100,000× *g* for 1 h, all the adhesion-promoting activity was lost from the supernatant but was quantitatively recovered in the pellet (Figure 1). This was the first indication that the ECM might be assembled from particulate material released from cells [10,11]. With the limited tools at hand in the late 1970s, it was next asked what is the nature and chemical structure of these particles that could be isolated by centrifugation and promoted cell adhesion? Based on this property, we called them adherons. The name “Klingons” was considered, but that might have infringed on Star Trek copyrights.

### 2.1. The Structure and Composition of Adherons

The size and heterogeneity of exosomes vary considerably depending upon the method of isolation, how the size is measured, and the source of the exosomes [12]. For our studies on adherons, the particles were isolated from serum-free culture medium using the identical method as described above essentially, and that is still used today to isolate exosomes. The serum-free conditioned medium was spun at low speed to remove cells and cellular debris followed by 2–3 h at 100,000× *g*, followed by washing the pellet twice by centrifugation [9,10]. For further purification and confirmation of homogeneity, the adherons were centrifuged on a 5 to 10% sucrose gradient for 20 h [9]. This method is essentially the same as that originally used to purify exosomes from the conditioned medium of reticulocytes [13] except that the reticulocyte study used a sizing column rather than a sucrose gradient. The purity of our adherons was confirmed by electron microscopy, but unfortunately, using a technique that did not stain cell membranes [14].

Our first manuscripts describing the chemical composition of adherons were published in 1980 [9,10]. At that time, proteomics, as we know it today, was not available, so most macromolecular compositions were studied using radioactive substrates that could be incorporated into proteins, sugars, etc. It was shown that skeletal muscle myoblasts secrete adherons that contain hyaluronic acid, chondroitin sulfate, heparan sulfate, and additional, undefined glycosoaminoglycans (GAGs) [9,10]. Some specific proteins were also identified in myoblast adherons, including fibronectin and collagen, similar to what has since been reported for some exosomes [15]. Importantly, we found that while adherons from skeletal muscle cells inhibited the adhesion of PC12 sympathetic nerve-like cells to culture dishes, adherons from smooth muscle, which is normally innervated by sympathetic nerves, promoted cell–substrate adhesion, suggesting a cell type specificity of adheron composition [16]. Further analysis suggested that this was due to differences in the GAGs that were present in the adherons as both fibronectin and collagen were found at similar levels in skeletal and smooth muscle adherons. Based on these results, we decided to focus further studies on the GAGs.

We also turned to chick neural retina cells since they had previously been shown to release material that promoted adhesion and were considered a model for studying brain development [17]. Similar to the skeletal and smooth muscle cell adherons, the chick neural retina adherons contained both proteins and GAGs [14], and an antiserum made against the adherons reduced cell–substratum adhesion of the cells. Further studies showed that the interaction between chick neural retina cells and their adherons was blocked by heparin and heparan sulfate [14]. Moreover, an antibody to a heparan sulfate proteoglycan (HSPG) isolated from the neural retina cells completely blocked adheron-mediated cell–substratum adhesion as well as cell–cell adhesion [14,18]. Additional data showed that HSPG was one of the cell surface receptors for adherons on neurons [18]. It was recently shown that HSPG is also a cell surface receptor for exosomes in a variety of cell types [19]. Thus, these data suggested the existence of an HSPG-mediated adhesion between adherons and cells that involved a high molecular weight protein that we speculated was fibronectin since HSPG binds fibronectin and exosomes contain fibronectin. This hypothesis was recently supported by a study that showed that exosomes bind to heparin sulfate via fibronectin on the cell surface [15]. In addition, adherons themselves also contain HSPG, which contributes to the promotion of cell–substratum adhesion [18]. Similar to the results with the PC12 cells, chick retinal cells did not attach to adherons isolated from skeletal muscle cells [18], further supporting the cell type-specificity of this process [18].

### 2.2. Isolation and Cloning of a Neurotrophic Factor from Adherons

Further studies identified two proteins in chick neural retina adherons that bind to HSPG on the cell surface. One, as noted above, was a large fibronectin-like molecule, which was later identified as N-CAM [20], and the other was a 20 kD protein isolated based on its ability to stimulate cell–substratum adhesion [21]. This protein was named purpurin because when SDS polyacrylamide gels containing the protein were stained with silver, the purpurin band turned bright purple. Purpurin stimulates neural retina cell–substratum adhesion by its interaction with heparan sulfate but not other proteoglycans and prolongs the survival of neural retina cells in culture, but not of cortical neurons [21]. Purpurin is found in the retina, but not other tissues. It was initially shown that purpurin has amino acid sequence homology to serum retinol-binding protein, but the serum protein is larger in size and does not stain purple on SDS polyacrylamide gels [22]. Both proteins, however, bind retinol. Purpurin also supports the survival of chick ciliary ganglion neurons, showing that it is also a bona fide neurotrophic factor [22].

With the more widespread introduction of molecular cloning in the 1980s, purpurin was eventually cloned and expressed in mammalian cells [23]. Consistent with the earlier protein data, purpurin RNA was found in the retina, but not brain, heart, or liver. Purpurin is highly concentrated between the outer segments of the photoreceptor cells, where it is synthesized. Given its 50% sequence homology with serum retinol-binding protein, it likely plays a role in the transport of retinol across the interphotoreceptor matrix. Purpurin is also involved in promoting neurite outgrowth and regeneration in the goldfish retina [24,25] and optic nerve regeneration [26]. Moreover, purpurin is a key molecule for cell differentiation during the development of zebrafish retina [27,28,29]. Purpurin is a member of the lipocalin protein family, structurally similar extracellular molecules that bind hydrophobic molecules and transport them between cells. Purpurin is also the name of an anthraquinone red/yellow dye found in madder plants that is widely studied in the context of drug discovery [30].

My work on adherons came to an abrupt end in 1985 when during a promotion hearing, I was told that adherons were artifacts and that if I wished to stay at Salk, I should stop working on them. For reasons that are still not clear to me, I chose to stay in La Jolla. This was the biggest mistake of my career and clearly shows that although I knew that they were real, I was not sufficiently committed to their study to relocate. Ironically, that was about the time that the field of exosome research was just starting to take off.

### 2.3. Adherons vs. Exosomes

While we discovered adherons entirely based on our cell adhesion assay with serum-free cell-conditioned medium, exosomes were first identified based on tracking transferrin receptors in reticulocytes [31]. Harding, Heuser, and Stahl were studying transferrin receptors (TRs) on reticulocyte plasma membranes and showed that they were endocytosed, and appeared in various intracellular membrane-bound compartments, including endosomes [31]. The TRs were then recycled back to the plasma membrane and ultimately released into the extracellular space indicating that TRs and possibly other reticulocyte proteins might be extruded by exocytosis [31]. However, there was no discussion of the structure of the extracellularly released TRs, although the assumption may have been that they were in membrane vesicles.

In 1983, Pan and Johnstone published a paper that used reticulocytes and radioactive anti-TR antibodies [13]. They showed that TRs co-migrate with extracellular vesicles that were isolated in a manner identical to adherons by centrifugation at 100,000× *g* and argued that there was a selective externalization of the TR-antibody complex in vesicles. These vesicles were heterogeneous in size but appeared to have a plasma membrane [13]. Instead of using a sucrose gradient to purify the TR-antibody complex further, they used size exclusion chromatography. During the next few years, the Johnstone lab focused on the extracellular particles from reticulocytes and named these particles exosomes in 1987 [32]. It was originally thought that exosomes were only associated with reticulocyte maturation and not found in other cell types [32], but during the last 30 years, it has been shown that essentially all cells secrete extracellular particles that can be recovered by centrifugation at 100,000× *g* for 2 h, as was first described for adherons [9,10,12,33]. The size of these extracellular vesicles varies, and while there have been attempts to classify them based on size, their composition and function are frequently not distinguishable, although exosomes are generally considered the smaller of the group [32].

Since the original descriptions of adherons and exosomes, there have been thousands of publications on them. For the remainder of this review, I will briefly return to the area of my initial focus on adherons and a subject that has not been well studied in the context of exosomes, their involvement in cell adhesion, and the ECM in the context of the brain.

### 2.4. Exosomes, Adhesion, and the Brain ECM

**Figure 2:**
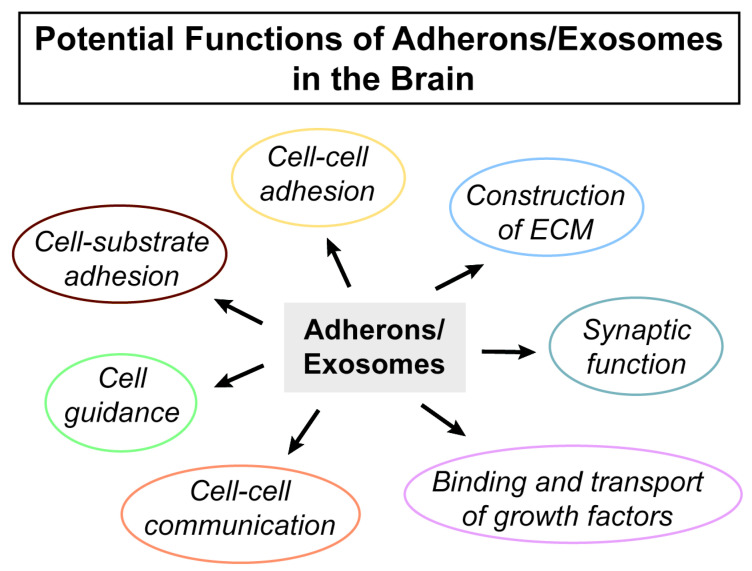
Potential functions of adherons/exosomes in the brain.

The ECM surrounding neurons and glia makes up about 20% of the brain’s mass, but there is much less information about the structure and function of the ECM than the cells that are embedded in it. The best-studied aspect of brain ECM is the perineuronal nets (PNNs) that surround a subset of inhibitory interneurons [34]. The structure of the PNN and ECM is modulated in part by matrix metalloproteases and linked to various aspects of learning, memory, and psychiatric disorders [34]. However, very little is known about how the ECM is made and specifically the role of exosomes in its biogenesis. It is likely that exosomes and their HSPGs play a critical role in the construction of the ECM surrounding neurons and glia that dynamically affects their function (Figure 2). Since the ECM and HSPGs are clearly involved in guiding cell migration [35], providing adhesion loci for cells [36] and enabling cellular communication [37,38], this suggests a more important role for exosomes in central nervous system (CNS) function than is currently realized.

Another possible role for exosome HSPGs is at the synapse [39,40]. Amyloid precursor protein (APP) is the precursor to Aβ and a single span membrane protein. Many years ago, we showed APP is cleaved from the membrane generating a non-amylogenic secreted protein [41]. It also strongly binds heparan [42], and growth factors such as fibroblast growth factor (FGF) increase the rate of APP secretion [43], and APP is associated with ECM [44]. Importantly, APP is found in brain exosomes [45]. Based on some of this information, we argued that APP at the synapse is exocytosed during excitation, thus facilitating and perhaps stabilizing synapses [39].

Finally, essentially all CNS growth factors, such as FGF, NGF, GDNF, and brain-derived neurotrophic factor (BDNF), are small basic proteins that bind HSPG. Thus, they have the potential to be found in exosomes, and this has indeed been seen in several studies [46,47,48]. Importantly, exosomes could provide a safe mechanism for transporting these molecules between cells and, if exosomes are incorporated into the ECM, then this would also be a way to keep the growth factors in the vicinity of the cells that secrete them.

## 3. Conclusions

Clearly, the field of exosomes has exploded since their first description in the early 1980s. While much of the work has been on exosomes derived from cells that comprise peripheral tissues, they clearly play an important role in brain function as well. However, while their role as potential biomarkers of disease has received a good deal of attention [38,49,50], an understanding of their role in normal brain physiology has lagged behind. I hope that this short review describing their early discovery and characterization as adherons will promote more interest in this important and understudied area of research.

## Abbreviations

BDNF	Brain-derived neurotrophic factor
CNS	Central nervous system
ECM	Extracellular matrix
FGF	Fibroblast growth factor
GAG	Glycosaminoglycan
GDGF	Glial-derived growth factor
HSPG	Heparin sulfate proteoglycan
NGF	Nerve growth factor
PNN	Perineuronal net
SAM	Substrate-attached material
TR	Transferrin receptor
